# A new toad species of the genus *Brachytarsophrys* Tian & Hu, 1983 (Anura, Megophryidae) from Guizhou Province, China

**DOI:** 10.3897/BDJ.10.e79984

**Published:** 2022-02-18

**Authors:** Shize Li, Jing Liu, Guiping Yang, Gang Wei, Haijun Su

**Affiliations:** 1 Moutai Institute, Renhuai, China Moutai Institute Renhuai China; 2 Guiyang College, Guiyang, China Guiyang College Guiyang China; 3 Guizhou University, Guiyang, China Guizhou University Guiyang China

**Keywords:** Megophryidae, new taxon, phylogenetic analysis, morphology

## Abstract

**Background:**

The toads of the genus *Brachytarsophrys* Tian & Hu, 1983 are distributed in southern China, Myanmar, Vietnam, Laos and northern Thailand. Seven species of the genus have been recognised, of which five of them are known from China so far.

**New information:**

*Brachytarsophrysqiannanensis* sp. nov., a new species of the short-legged toad genus is here described from southern Guizhou Province, China. Diagnostic characters of the new species are illustrated and comparisons with its congeners are provided. Its validity is also affirmed by its distinct mitochondrial gene sequence divergence with all congeners and its monophyly recovered in the mitochondrial gene-based phylogenetic analyses.

## Introduction

The short-legged toad genus *Brachytarsophrys* Tian & Hu, 1983 occurs widely in southern China, Myanmar, Vietnam, Laos and northern Thailand (Frost 2022). The generic taxonomy of this group has been controversial for a long time. Some studies (Dubois 1987, Duellman 1993, Dubois and Ohler 1998, Xu 2005, Mahony et al. 2017) considered this group as a subgenus of *Megophrys* Kuhl & Van Hasselt, 1822, but most recent studies still retained it as a genus mainly based on its distinct morphology, special ecological traits and the independent phylogenetic position (e.g. Fei et al. 1990, Ye and Fei 1992, Zhao and Adler 1993, Rao and Yang 1997, Fei et al. 2009, Pyron and Wiens 2011, Fei and Ye 2016, Chen et al. 2017, Li et al. 2020, Luo et al. 2021, Lyu et al. 2021, Tapley et al. 2021, Frost 2022).

The genus *Brachytarsophrys* currently contains seven species and Li et al. (2020) suggested they are divided into two groups, namely the *Brachytarsophryscarinense* group and the *Brachytarsophrysfeae* group. The *Brachytarsophryscarinense* group contains *B.carinens* (Boulenger, 1889) and *B.intermedia* (Smith, 1921) and the *Brachytarsophrysfeae* group contains *B.feae* (Boulenger, 1887), *B.chuannanensis* Fei, Ye and Huang, 2001, *B.platyparietus* Rao and Yang, 1997, *B.popei* Zhao, Yang, Chen, Chen & Wang, 2014 and *B.orientalis* Li, Lyu, Wang & Wang, 2020. Some phylogenetic studies indicated that the species diversity of *Brachytarsophrys* has been underestimated (e.g. Li et al. 2020, Lyu et al. 2021).

During field surveys in Libo County, Qiannan Autonomous Prefecture, Guizhou Province, China in 2021, we collected a series of *Brachytarsophrys* toads. Molecular phylogenetic analyses and morphological comparisons supported it as an undescribed species of *Brachytarsophrys*. We describe it herein as a new species.

## Materials and methods

**Figure 1.**
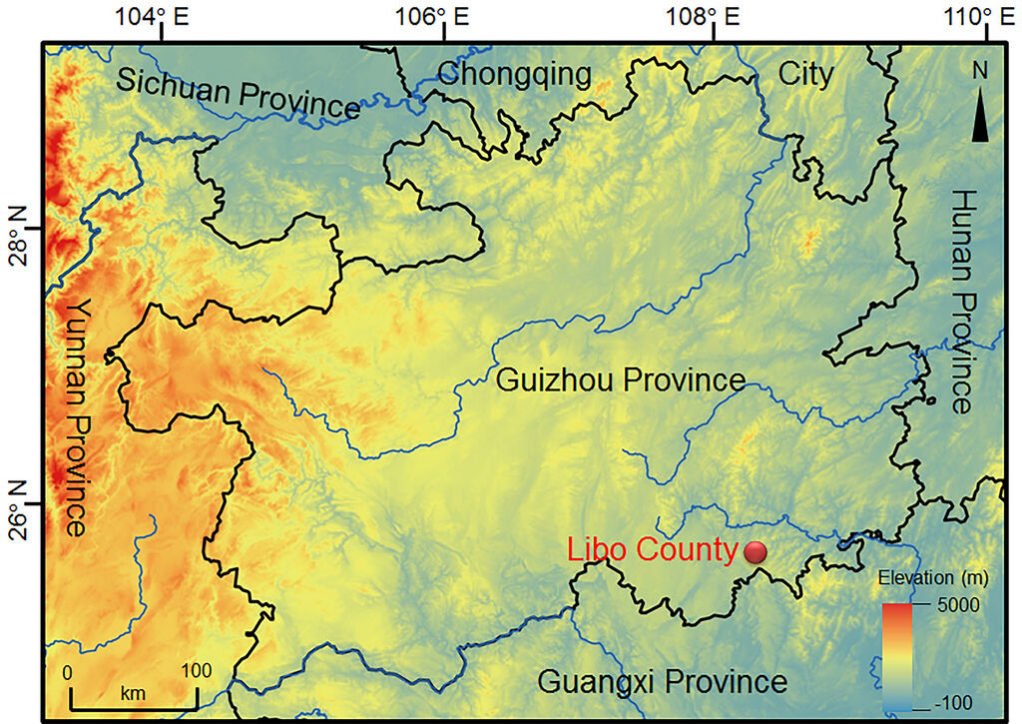
Geographical location of the type locality of *Brachytarsophrysqiannanensis* sp. nov., Libo County, Guizhou Province, China.

One adult male and three adult females of the *Brachytasophrys* sp. were collected from Libo County (**LB**), Guizhou Province, China (see Suppl. material 1, Fig. 1). In the field, the toads were euthanised using isoflurane and the specimens were fixed in 75 % ethanol. Tissue samples were taken and preserved separately in 99 % ethanol prior to fixation. The specimens were deposited in Chengdu Institute of Biology（**CIB)**, Chinese Academy of Sciences (**CAS**).

All adult specimens of the *Brachytasophrys* sp. were measured. The terminology and methods followed Fei and Ye (2016) and Li et al. (2020). Measurements were taken with a dial caliper to 0.1 mm. Sixteen morphometric characters of adult specimens were measured: eye diameter (ED, distance from the anterior corner to the posterior corner of the eye); foot length (FL, distance from distal end of shank to the tip of Toe IV); head length (HDL, distance from the tip of the snout to the articulation of jaw); maximum head width (HDW, greatest width between the left and right articulations of jaw); internasal distance (IND, minimum distance between the inner margins of the external nares); interorbital distance (IOD, minimum distance between the inner edges of the upper eyelids); length of lower arm and hand (LAL, distance from the elbow to the distal end of the Finger IV); lower arm width (LW, maximum width of the lower arm); snout-vent length (SVL, distance from the tip of the snout to the posterior edge of the vent); snout length (SL, distance from the tip of the snout to the anterior corner of the eye); length of foot and tarsus (TFL, distance from the tibiotarsal articulation to the end of the Toe IV); thigh length (THL, distance from vent to knee); tibia length (TL, from the outer surface of the flexed knee to the heel); maximal tibia width (TW); maximal tympanum diameter (TYD); upper eyelid width (UEW, greatest width of the upper eyelid margins measured perpendicular to the anterior-posterior axis). The location of the web on the phalange articulation was designated as follows: - (distal part of phalange articulation); none (middle part of phalange articulation); + (proximal part of phalange articulation); ++ (lower part of phalange articulation) followed the protocol described by Savage (1975) and Li et al. (2020).

Sex was determined by secondary sexual characters, i.e. the presence of vocal sac and nuptial pads/spines in male (Fei and Ye 2016).

The *Brachytasophrys* sp. was also compared with all other *Brachytarsophrys* species, based on morphological characteristics. Comparative morphological data were obtained from literature for *B.carinense* (Boulenger 1889, Li et al. 2020), *B.chuannanensis* (Fei and Ye 2001, Li et al. 2020), *B.feae* (Boulenger 1887, Li et al. 2020), *B.intermedia* (Smith 1921, Li et al. 2020), *B.orientalis* (Li et al. 2020), *B.platyparietus* (Rao and Yang 1997, Li et al. 2020) and *B.popei* (Zhao et al. 2014, Li et al. 2020).

The advertisement calls were recorded in the field on 6 August 2021 in Libo County, Qiannan Autonomous Prefecture, Guizhou Province, China. It was recorded in the stream at ambient air temperature of 18.0°C and air humidity of 80%. SONY PCM-D50 digital sound recorder was used to record within about 50 cm of the calling individual. The sound files in wave format were resampled at 48 kHz with sampling depth 24 bits. PRAAT 6.0.27 (Boersma 2001) was used to obtain the sonograms and waveforms (window length = 0.005s). Raven pro 1.5 software (Bioacoustics Research Program 2013) was used to quantify the acoustic properties (window size = 256 points, fast Fourier transform, Hanning window). Terminology of advertisement call analyses and description followed Köhler et al. (2017). Ambient temperature was taken by a digital hygrothermograph.

Four specimens of the *Brachytasophrys* sp. were included in the molecular analyses (Table 1). Total DNA was extracted using a standard phenol-chloroform extraction protocol (Sambrook et al. 1989). Three fragments of the mitochondrial 16S rRNA (16S), cytochromeoxidase subunit I (COI) and cytochrome b (Cytb) genes were amplified. For 16S, the primers P7 (5'-CGCCTGTTTACCAAAAACAT-3') and P8 (5'-CCGGTCTGAACTCAGATCACGT-3') were used following Simon et al. (1994); for COI, Chmf4 (5'-TYTCWACWAAYCAYAAAGAYATCGG-3') and Chmr4 (5’-ACYTCRGGRTGRCCRAARAATCA-3’) were used following Che et al. (2012) and for Cytb, PFGlu14140L (5'-GAAAAACCACTGTTGTHHYTCAACTA-3') and PFThr15310 (5'-CGGYTTACAAGACCGRTGCTTT-3') were used following Zhang et al. (2013). Gene fragments were amplified under the following conditions: an initial denaturing step at 95 °C for 4 min; 36 cycles of denaturing at 95 °C for 30 s, annealing at 54 °C (for 16S)/49 °C (for COI)/50 °C (for Cytb) for 40 s and extending at 72 °C for 70 s. Sequencing was conducted using an ABI3730 automated DNA sequencer in Shanghai DNA BioTechnologies Co., Ltd. (Shanghai, China). New sequences were deposited in GenBank (for GenBank accession numbers see Table 1).

For molecular analyses, the available sequence data for *Brachytarsophrys* were downloaded from GenBank (Table 1), primarily from previous studies (Chen et al. 2017, Li et al. 2020). For phylogenetic analyses, corresponding sequences of one *Atympanophrysshapingensis* Liu 1950 and one *Panophrysomeimontis* Liu 1950 were also downloaded (Table 1) and used as outgroups according to Chen et al. (2017). Sequences were assembled and aligned using the Clustalw module in BioEdit v.7.0.9.0 (Hall 1999) with default settings. For phylogenetic analyses of mitochondrial DNA, the dataset was concatenated with 16S, COI and Cytb gene sequences. To avoid under- or over-parameterisation (Lemmon and Moriarty 2004, McGuire et al. 2007), the best partition scheme and the best evolutionary model for each partition were chosen for the phylogenetic analyses using PARTITIONFINDER v. 1.1.1 (Robert et al. 2012). In this analysis, 16S gene and each codon position of protein-coding genes were defined and Bayesian Inference Criteria was used. As a result, the analysis suggested that the best partition scheme is 16S gene/each codon position of protein-coding genes and selected GTR + G + I model as the best model for each partition. Phylogenetic analyses were conducted using Maximum Likelihood (ML) and Bayesian Inference (BI) methods, implemented in PhyML v. 3.0 (Guindon et al. 2010) and MrBayes v. 3.12 (Ronquist and Huelsenbeck 2003), respectively. For the ML tree, branch supports were drawn from 10,000 non-parametric bootstrap replicates. In BI, two runs each with four Markov chains were simultaneously run for 50 million generations with sampling every 1,000 generations. The first 25% trees were removed as the “burn-in” stage followed by calculations of Bayesian posterior probabilities and the 50% majority-rule consensus of the post burn-in trees sampled at stationarity, bootstrap supports (BS) and Bayesian Posterior Probabilities (BPP) are shown at the nodes. Finally, mean genetic distance between samples in this study, based on uncorrected *p*-distance model, was estimated using MEGA v. 6.06 (Tamura et al. 2013) with the pairwise deletion setting for the Gap/Missing Data.

## Data resources

All the sequences in this study were retrieved from GenBank and the accession numbers of the newly-determined sequences in this study are shown in Table 1.

## Taxon treatments

### Brachytarsophrys qiannanensis

Li, Liu, Yang, Wei, & Su sp. n.

1C96298F-ABE7-5CCC-850D-EEF593050D6E

92662CAA-955E-41B2-B991-8905E6E65FFA

#### Materials

- **Type status:** Holotype. **Occurrence:** recordedBy: Jing Liu; individualID: CIB LB20210806054; individualCount: 1; sex: male; lifeStage: adult; **Taxon:** scientificName: *Brachytarsophrysqiannanensis*; kingdom: Animalia; phylum: Chordata; class: Amphibia; order: Anura; family: Megophryidae; genus: Brachytarsophrys; **Location:** higherGeography: South-western China; country: China; stateProvince: Guizhou Province; county: Libo County; municipality: Qiannan Autonomous Prefecture; locality: Changniu Village; verbatimElevation: 1190; verbatimCoordinates: 25.572492°N, 108.274189°E; georeferenceSources: georeferenceSources; **Identification:** identifiedBy: Shize Li; **Event:** eventDate: 06/08/2021; **Record Level:** type: Even
- **Type status:** Paratype. **Occurrence:** recordedBy: Jing Liu; individualID: CIB LB20210806055-57; individualCount: 3; sex: 3 females; lifeStage: adul; **Taxon:** scientificName: *Brachytarsophrysqiannanensis*; kingdom: Animalia; phylum: Chordata; class: Amphibia; order: Anura; family: Megophryidae; subgenus: Brachytarsophrys; **Location:** higherGeography: South-western China; country: China; stateProvince: Guizhou Province; county: Libo County; municipality: Qiannan Autonomous Prefecture; locality: Changniu Village; verbatimCoordinates: 25.572492°N, 108.274189°E; georeferenceSources: Google Earth; **Identification:** identifiedBy: Shize Li; **Event:** eventDate: 06/08/2021; **Record Level:** type: Even

#### Diagnosis

**Figure 2.**
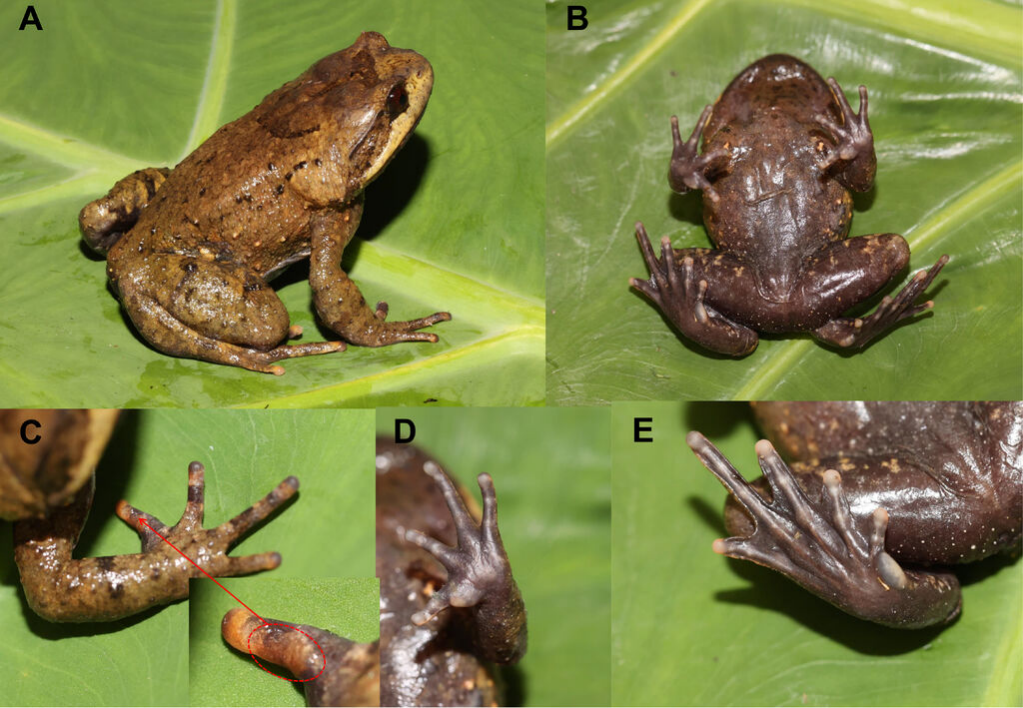
Photos of the holotype CIB LB20210806054 of *Brachytarsophrysqiannanensis* sp. nov. in life. **A** dorsal view; **B** ventral view; **C** dorsal view of hand (insert: the nuptial pad on the dorsal surface of the first finger); **D** ventral view of hand; **E** ventral view of foot.

Morphometric measurements for specimens examined are given in Table 2 and Suppl. material 1. See Fig. 2A-E for dorsal and ventral view of body, dorsal and ventral view of hand and ventral view of foot.

*Brachytarsophrysqiannanensis* sp. nov. could be distinguished from its congeners by a combination of the following morphological characters: (1) body size small (SVL 70.1 mm in male and 80.1 – 84.9 mm in females); (2) tongue pyriform, feebly notched posteriorly; (3) tibiotarsal articulation reaching to commissure of jaw when leg stretched forward; (4) toes about one third to two thirds webbed in males; (5) male with a single subgular vocal sac and a brown nuptial pad present on the dorsal surface of the first finger.

##### Description of holotype

An adult male, SVL 70.1 mm; head enormous, extremely depressed, about 1.7 times as broad as long; snout short, rounded in dorsal view, slightly protruding beyond margin of lower jaw; canthus rostralis indistinct; loreal region very oblique, slightly concave; nostril closing to the tip of snout; tympanum not obvious; eye large, eye diameter 31 % of head length; maxillary teeth present, vomerine teeth present on two vomerine ridges; tongue pyriform, notched posteriorly.

Fore-limbs short and moderately robust, the length of lower arm and hand 42 % of SVL; fingers rather short without web, relative finger lengths: I < II < V < III; tips of digits round, feebly dilated; lateral fringes absent; metacarpal tubercle two, inner one significantly enlarged, outer one slightly enlarged.

Hind-limbs relatively short and robust, heels not meeting when thighs are positioned at right angles to the body, tibiotarsal articulation reaching to commissure of jaw when leg stretched forward; tibia length longer than thigh length; relative toe lengths I < II < V < III < IV; tips of toes round, slightly dilated; toes about one third to two thirds webbed and lateral fringe wide, the webbing formula is I (1) - (2^+^) II (2-) - (3-) III (2½) - (4-) IV (4^++^) - (2-) V; inner metatarsal tubercle oval-shaped; outer metatarsal tubercle absent.

Dorsal skin rough, several conical tubercles scattered on flank of trunk, dorsum of body and limbs; upper eyelid with several tubercles and one enlarged to form horn; tubercles on the dorsum forming a U-marking on the anterior dorsum; a dark brown streak on dorsum of head and between the eyes; supratympanic fold distinct, from posterior corner of eye to a level above the shoulder.

Ventral surface smooth; pectoral gland distinct, closer to axilla than to mid-ventral line; rear of thigh with a small femoral gland, around which densely arranged granules forming a granular patch.

**Colouration of holotype in life**: Dorsal brown, a dark brown streak on dorsum of head and between the eyes; dark tubercles present on the dorsum, forming a U-marking, some dark tubercles scattered on the shoulder and posterior dorsum, flank of body scattered with some light brown tubercles; upper lip light brown; tympanic region brown; dorsal digits with dark brown transverse bands and three transverse skin ridges on the dorsal shank and thigh; ventral surface brown-black, pectoral gland yellowish; several yellowish spots on two sides of belly; lower surface of digits purple-grey; webs, palms and soles purple-grey; tip of digits, two metacarpal tubercle and inner metatarsal tubercle grey-white; nuptial pad brown; the tubercles at upper eyelid yellowish; pupils black; iris brownish.

**Figure 3.**
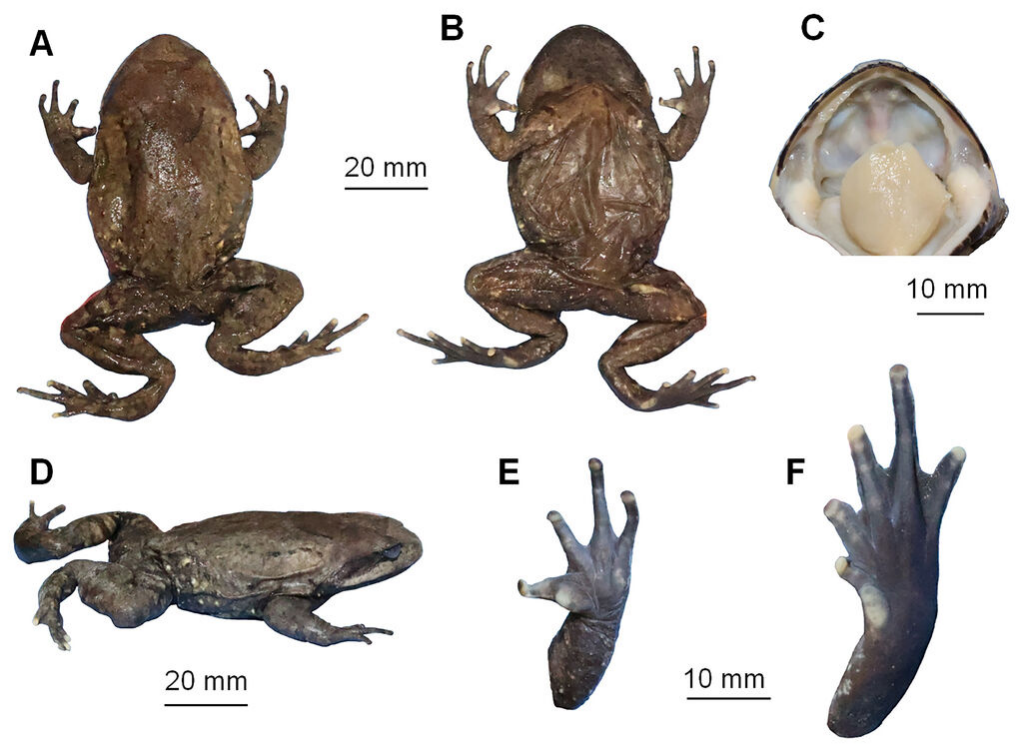
The holotype specimen CIB LB20210806054 of *Brachytarsophrysqiannanensis* sp. nov. in preservative. **A** dorsal view; **B** ventral view; **C** view of oral cavity; **D** lateral view; **E** ventral view of hand; **F** ventral view of foot.

**Colouration of holotype in preservation**: Colour of dorsal surface fades to pale brown; ventral surface brown; the posterior of ventral surface of body; tip of digits, two metacarpal tubercles and inner metatarsal tubercle grey-white fades to white (Fig. 3).

**Figure 4.**
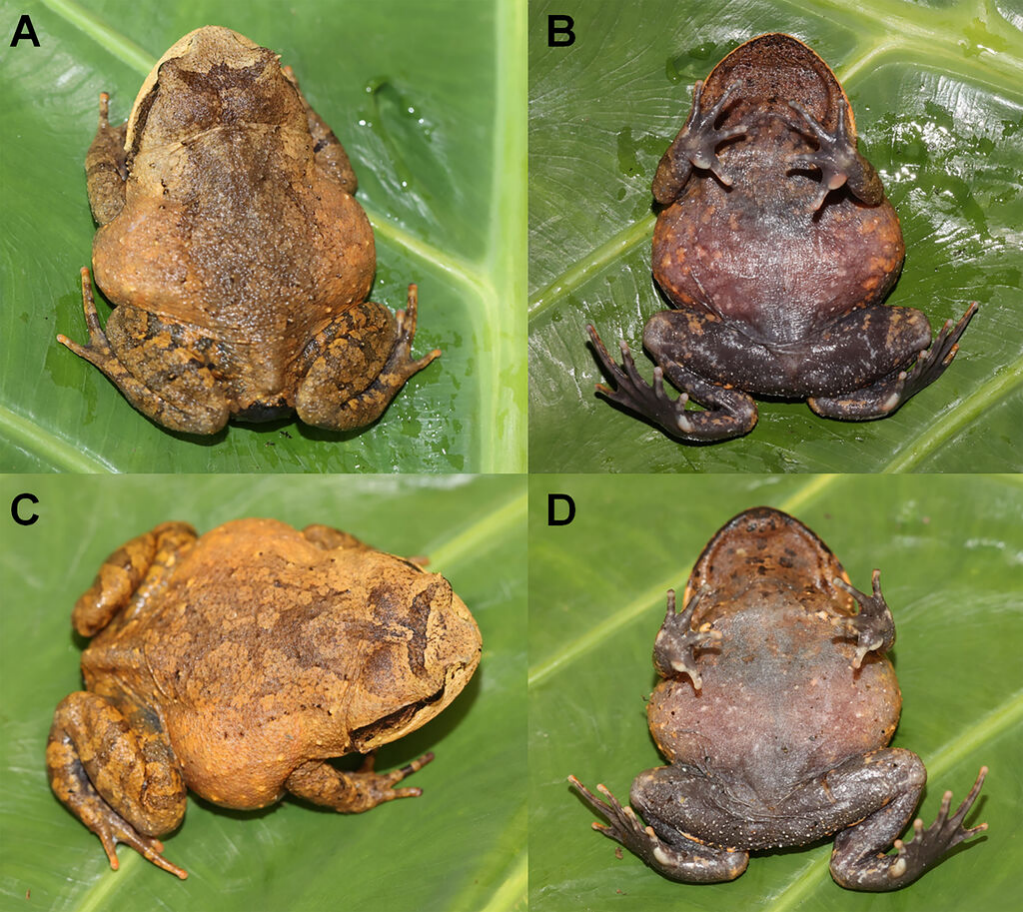
Colour variation *Brachytarsophrysqiannanensis* sp. nov. in life **A** dorsal view of the female specimen CIB LB20210806055; **B** ventral view of the female specimen CIB LB20210806055; **C** dorsal view of the specimen the female specimen CIB LB20210806055; **D** ventral view of the female specimen CIB LB20210806056.

**Variation**: Measurements of the type series are shown in Suppl. material 1. In this new species, the females had larger bodies than male (Table 2). In life, the diagnostic morphological characters of all paratypes were identical to those of the holotype (Fig. 4). However, colouration and stripe patterns differed amongst individuals. In CIB LB20210806055, the brown patches on dorsum are irregular (Fig. 4A), in the ventral surface, some dark tubercles on the throat and the tubercles on the thigh are white (Fig. 4B); in CIB LB20210806056, the brown patches forming an X-marking on the dorsum (Fig. 4C) and the colouration of ventral surface is lighter (Fig. 4D).

**Secondary sexual characters**: The adult male with a single subgular vocal sac and brown nuptial pad present on the dorsal surface of the first finger (Fig. 2C).

**Figure 5.**
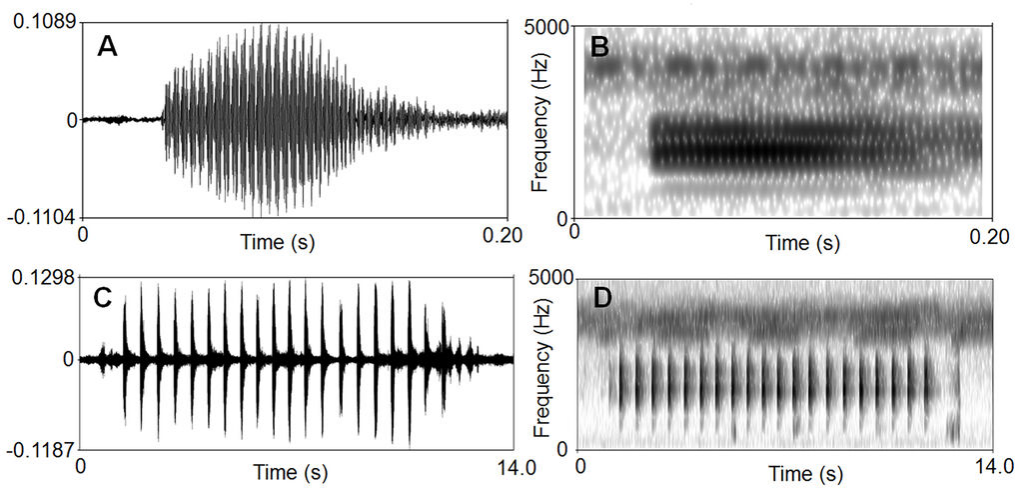
Visualisation of advertisement calls of *Brachytarsophrysqiannanensis* sp. nov. **A** waveform showing one note; **B** sonogram showing one note; **C** waveform showing 20 notes of one call; **D** sonogram showing 20 notes of one call (A and B are the same note, C and D are the same call).

**Advertisement call**: The call description is based on recordings of the holotype CIB LB20210806054 (Fig. 5), calling from beneath a large stone in a streamlet and the ambient air temperature was 18.0°C. Each call consists of 16 – 20 (mean 17.3.5 ± 2.3, n = 3) notes. Call duration was 7690–11330 ms (mean 9068 ± 1974, n = 3). Call interval was 10980–15670 ms (mean 13325 ± 3316, n = 2). Each note had a duration of 129–348 ms (mean 249 ± 36, n = 52) and the intervals between notes 180–395 ms (mean 269 ± 42, n = 49). Amplitude modulation within the note was apparent, beginning with moderately high energy pulses, increasing slightly to a maximum by approximately mid-note and then decreasing towards the end of each note. The average dominant frequency was 1740 ± 168 Hz (1640 – 2330 Hz, n = 3).

##### Comparisons

Comparative data of *Brachytarsophrysqiannanensis* sp. nov. with other seven recognised congeners of *Brachytarsophrys* are given in Suppl. material 2

*Brachytarsophrysqiannanensis* sp. nov. differs from *B.orientalis* by having a smaller body size , SVL 70.1 mm in male and 80.1 – 84.9 mm in females (vs. 76.8 – 82.7 mm in males, n = 7 and 88.6 mm in female); different webbing formula I (1) - (2^+^) II (2-) -(3-) III (2½) - (4-) IV (4^++^) - (2-) V in male and I (1½) - (2^+^) II (2-) - (3^+^) III (2½) - (4) IV (4^++^) - (2) V in female (vs. I (1½) - (2) II (1½) - (3) III (2½) - (4) IV (4) - (2) V in male and I (2) - (2^+^) II (1⅔) - (3) III (3-) - (4) IV (4^+^) -(2½) V in female); the male specimen with a brown nuptial pad present on the dorsal surface of the first finger (vs. dark brown nuptial pads present on the dorsal surface of the first two fingers)(Li et al. 2020).

*Brachytarsophrysqiannanensis* sp. nov. differs from *B.popei* by having tongue feebly notched posteriorly (vs. deeply notched behind); nuptial pad of the male without spines on the dorsal surface of the first finger (vs. with black nuptial spines present on the dorsal bases of the first two fingers); webbing formula I (1) - (2^+^) II (2-) - (3-) III (2½) - (4-) IV (4^++^) - (2-) V in male and I (1½) - (2^+^) II (2-) - (3^+^) III (2½) - (4) IV (4^++^) - (2) V in female (vs. I (1½) - (2) II (1½) - (3) III (2½) - (3⅔) IV (3⅔) - (2) V in male and I (1½) - (2^+^) II (1½) - (3) III (2½) - (4-) IV (4-) - (2) V in female) (Zhao et al. 2014, Li et al. 2020, Suppl. material 2).

*Brachytarsophrysqiannanensis* sp. nov. differs from *B.platyparietus* by having a smaller size SVL 70.1 mm in male and 80.1 – 84.9 mm in three females (vs. 88.5 – 113.0 mm in males, n = 6 and 118.5 – 131.0 mm in female, n = 3); lateral fringes on toes narrow (vs. wide); brown nuptial pad without nuptial spines present on the dorsal surface of the first finger (vs. dark brown nuptial pads with black nuptial spines present on the dorsal bases of the first two fingers); webbing formula I (1) - (2^+^) II (2-) - (3-) III (2½) - (4-) IV (4^++^) - (2-) V in male and I (1½) - (2^+^) II (2-) - (3^+^) III (2½) - (4) IV (4^++^) - (2) V in female (vs. I (1½)-(2^+^) II (1½)-(3) III (2⅓)-(3⅔) IV(3⅔)-(2-) V in male and I (1½)-(2^+^) II (1½)-(3) III (2⅔)-(4-) IV (4-)-(2⅔) V) (Rao and Yang 1997, Li et al. 2020, Suppl. material 2).

*Brachytarsophrysqiannanensis* sp. nov. differs from *B.feae* by having a smaller body size in male, SVL 70.1 mm (vs. 78.5 – 94.9 mm in males, n = 5); tibiotarsal articulation reaching to commissure of jaw when leg stretched forward both in male and females (vs. reaching to shoulder in females); brown nuptial pad present on the dorsal surface of the first finger (vs. dark brown nuptial pads present on the dorsal bases of the first two fingers); webbing formula I (1) - (2^+^) II (2-) - (3-) III (2½) - (4-) IV (4^++^) - (2-) V in male (VS. I (2)-(2^++^) II (2-)-(3)III (2⅔)-(4) IV (4)-(2⅔) V) (Boulenger 1887, Li et al. 2020, Suppl. material 2).

*Brachytarsophrysqiannanensis* sp. nov. further differs from *B.feae* by having more notes of each call (16 – 20 notes vs. 4 – 5 notes); a longer call duration 7690–11330 ms (vs. 2256–35488 ms) and a higher dominant frequency 1640 – 2330 Hz (vs. 1378 Hz) (Wogan et al. 2004).

*Brachytarsophrysqiannanensis* sp. nov. differs from *B.chuannanensis* by having a smaller body size SVL 70.1 mm in male (vs. 91.4 – 109.4 mm in males, n = 12); the male with brown nuptial pad on dorsal surface of the first finger (vs. dark brown nuptial pads present on the dorsal bases of the first two fingers); lateral fringes on toes of male wider (one third as broad as distal toe phalanx vs. one fifth)(Fei and Ye 2001, Li et al. 2020, Suppl. material 2).

*Brachytarsophrysqiannanensis* sp. nov. from *B.carinense* by having a smaller body size SVL 70.1 mm in male and 80.1 – 84.9 mm in females (vs. 91.6 – 123.0 mm in males, n= 4 and 124.0 – 168.0 mm, n = 3); by the absence of dermal ridges on dorsum (vs. present); tibiotarsal articulation reaching to commissure of jaw when leg stretched forward (vs. reaching to axilla in females and angle of mouth in males); webbing formula I (1) - (2^+^) II (2-) - (3-) III (2½) - (4-) IV (4^++^) - (2-) V in male (VS. I (1½)-(2^++^) II (2)-(3^++^) III (3)-(4) IV(4^++^)-(2½) V) (Boulenger 1889, Li et al. 2020, Suppl. material 2).

*Brachytarsophrysqiannanensis* sp. nov. differs from *B.intermedia* by having a smaller body size (SVL 70.1 mm in male and 80.1 – 84.9 mm in females vs. 86.0 – 103.0 mm in males, n = 7 and 92.0 mm in female) and the absence of glandular folds on dorsum (vs. present) (Smith 1921, Li et al. 2020, Suppl. material 2).

#### Etymology

The specific name *qiannanensis* refers to the distribution of this species, Qiannan Autonomous Prefecture, the County to where the type locality of the species belongs. We propose the common English name “Qiannan short-legged toad” and Chinese name “Qian Nan Duan Tui Chan (黔南短腿蟾)”.

#### Distribution

*Brachytarsophrysqiannanensis* sp. nov. is known from the type locality, Libo County, Guizhou Province, China at elevations between 1100 – 1200 m a.s.l.

#### Ecology

**Figure 6.**
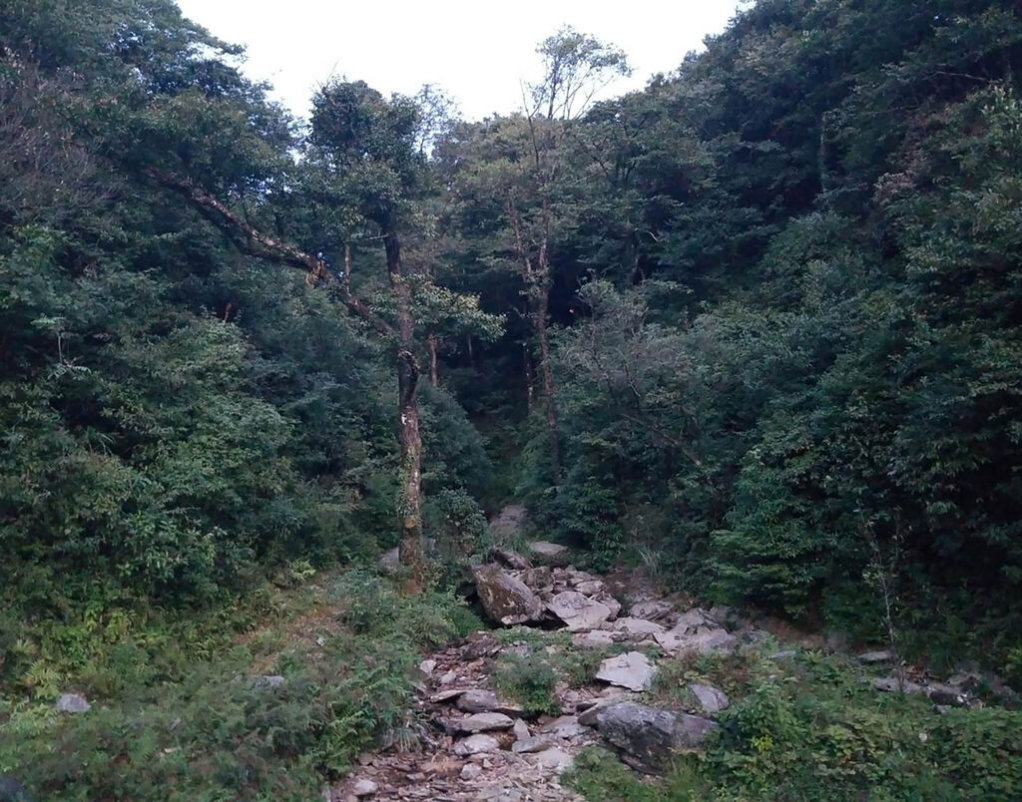
Habitat of *Brachytarsophrysqiannanensis* sp. nov. in the type locality, Libo County, Guizhou Province, China.

*Brachytarsophrysqiannanensis* sp. nov. inhabits a mountain stream (Fig. 6) covered by evergreen broadleaf forest, there being only a small amount of water on the surface of the stream. Advertisement call of males can be heard from beneath the rocks at night and the females were frequently found near large rocks.

## Analysis

**Phylogenetic analyses**

**Figure 7.**
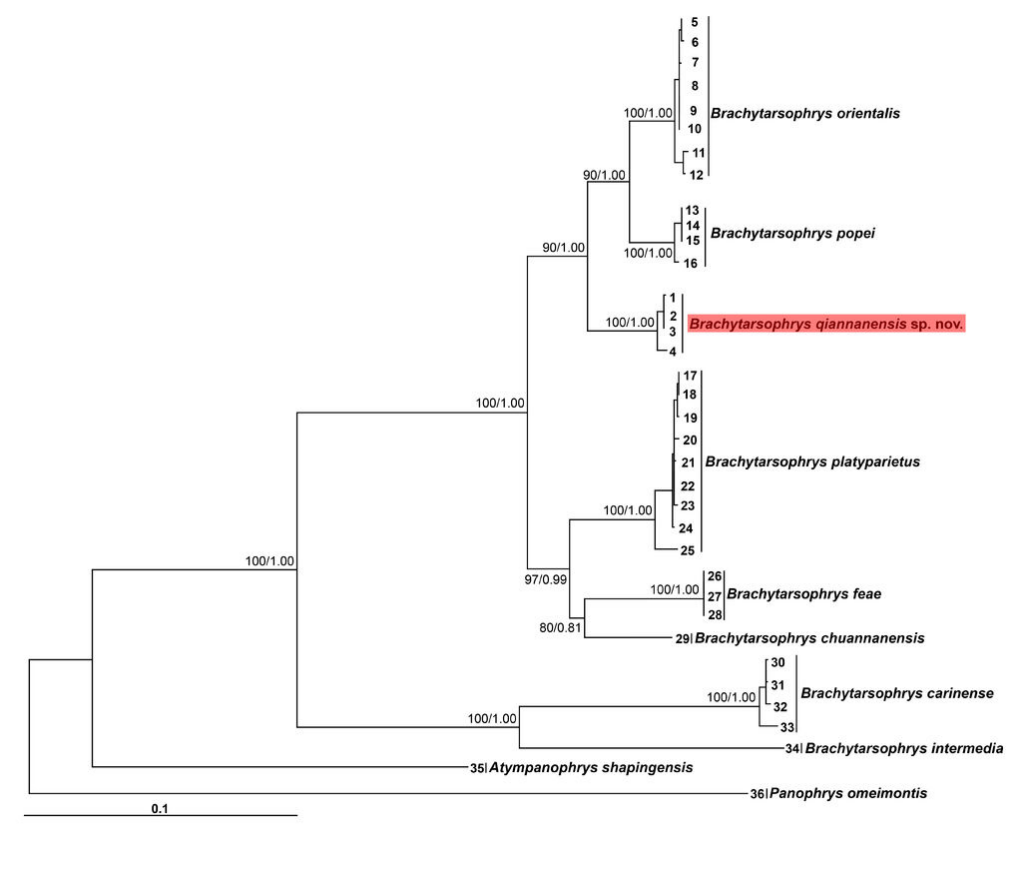
Phylogenetic tree reconstructed using Bayesian Inference (BI) and Maximum Likelihood (ML) methods, based on 16S, COI and Cytb genes. Values at the nodes correspond to BPP and BS. Samples 1–36 refer to Table 1.

Aligned sequence matrix of 16S + COI + Cytb contains 2061 bp. ML and BI trees of the mitochondrial DNA dataset presented almost consistent topology (Fig. 7). *Brachytarsophrysqiannanensis* sp. nov. was clustered into the *Brachytarsophrys* clade and grouped with *B.orientalis* and *B.popei* with high supported values (node supports in ML and BI: 90 and 1.00).

The mean genetic distances (*p*-distance) between *Brachytarsophrysqiannanensis* sp. nov. and its congeners was 2.1% on 16S (with *B.popei*), 4.9% on COI (with *B.orientalis*) and 6.6% on Cytb (with *B.orientalis*), these distances being much higher than those between some pairs of recognised congeners (Suppl. materials 3, 4, 5); for example, the COI *p*-distance was 4.3% between *B.orientalis* and *B.popei*, while the Cytb *p*-distance between *B.orientalis* and *P.popei* was 3.3%.

Molecular phylogenetic analyses showed that the population of *Brachytarsophrys* from Libo County, Qiannan Autonomous Prefecture, Guizhou Province, China is also distinct from its congeners.

## Discussion

The previous morphological studies indicated that, in the genus *Brachytarsophrys*, only *B.carinense* was recorded from Leishan and Anlong Counties of Guizhou Province (e.g. Wu et al. 1986, Fei et al. 2009, Fei et al. 2012). Lyu et al. (2014) further identified the population from Fanjing Mountain, Guizhou Province as *B.chuannanensis*, based on morphological comparisons. Based on molecular phyogenetic analyses and morphological comparisons, Li et al. (2020) suggested that *B.platyparietus* should be a valid species and populations in south-western China previously recognised as *B.carinense* should be re-identified as *B.platyparietus*, including the population from Fanjing Mountain, Guizhou Province. Moreover, Li et al. (2020) suggested that the populations from the western Hunan Province and north-western part of Guangxi Province, all adjoining Guizhou Province, belonged to *B.popei* and *B.orientalis*, respectively. Accordingly, it is inferred that the population of *Brachytarsophrys* from Anlong County in western Guizhou Province, near Yunnan Province, may be *B.platyparietus* and that from Leigong Mountain in south-eastern Guizhou Province, near the Hunan Province, may be *B.popei* or *B.orientalis*. In addition, the taxonomic assignments of the specimens from Fanjing Mountain, Guizhou Province are still doubtful because Li et al. (2020) reported that the specimen from Fanjing Mountain unexpectedly shared a common holotype on the COI gene with several specimens from the northern and central parts of Yunnan Province which is quite far from Fanjing Mountain in the eastern Guizhou Province. It is a pity that Li et al. (2020) did not provide more morphological and bioacoustics information of this specimen for comparisons with other species. Obviously, the taxonomic profiles of *Brachytarsophrys* in Guizhou Province are still unresolved and further investigations on this group should be conducted in the region.

*Brachytarsophrysqiannanensis* sp. nov. seems to be the smallest species (SVL 70.1 mm in male and SVL < 85 mm in females) in the genus *Brachytarsophrys*. Whether its niche characteristics promote the special morphology in this species maybe an interesting evolutionary question.

In recent years, more than 20 new amphibian species have been discovered in Guizhou Province, China (Frost 2022). However, during our frequent and extensive surveys in Guizhou Province from 2016 to 2021, we only found one adult male and three adult females of *Brachytarsophrysqiannanensis* sp. nov. This perhaps indicated that the population of the species in Guizhou Province is potentially small. Hence, further surveys are needed to evaluate the population status of the species.

## Supplementary Material

XML Treatment for Brachytarsophrys qiannanensis

C6F96A62-1E8F-5DA2-A424-1436328DBEC4 10.3897/BDJ.10.e79984.suppl1 Supplementary material 1 Measurements of the adult specimens of *Brachytarsophrysqiannanensis* sp. nov. Data type morphological Brief description Measurements of the adult specimens of *Brachytarsophrysqiannanensis* sp. nov. Units are in mm. See abbreviations for the morphological characters in Materials and Methods section. File: oo_623666.xlsx https://binary.pensoft.net/file/623666 Shize Li, Jing Liu, Guiping Yang, Gang Wei, HaiJun Su

4AC1AA56-22AA-51BD-B7A9-ACCD2E8C20AD 10.3897/BDJ.10.e79984.suppl2 Supplementary material 2 Diagnostic characters separating the new species from other species of *Brachytarsophrys* Data type morphological Brief description Diagnostic characters separating the new species described in this study from other species of *Brachytarsophrys* (/ = not available). File: oo_647524.xlsx https://binary.pensoft.net/file/647524 Shize Li, Jing Liu, Guiping Yang, Gang Wei, Haijun Su

2DB88606-62AB-5137-B4FD-4126A3BA15F9 10.3897/BDJ.10.e79984.suppl3 Supplementary material 3 Mean uncorrected genetic p-distance of the 16S gene between samples
Data type genomic Brief description Mean uncorrected genetic p-distance of the 16S gene between samples examined in this study. File: oo_645611.xls https://binary.pensoft.net/file/645611 Shize Li, Jing Liu, Guiping Yang, Gang Wei, Hainjun S

6C29665D-0CFB-55FB-923E-D9A1EA426FF6 10.3897/BDJ.10.e79984.suppl4 Supplementary material 4 Mean uncorrected genetic p-distance of the COI gene between samples Data type genomic Brief description Mean uncorrected genetic p-distance of the COI gene between samples examined in this study. File: oo_623667.xls https://binary.pensoft.net/file/623667 Shize Li, Jing Liu, Guiping Yang, Gang Wei, HaiJun Su

900BC8DA-F15D-5700-84C0-D1E9D76FBE3D 10.3897/BDJ.10.e79984.suppl5 Supplementary material 5 Mean uncorrected genetic p-distance of the Cytb gene between samples Data type genomic Brief description Mean uncorrected genetic p-distance of the Cytb gene between samples examined in this study. File: oo_623668.xls https://binary.pensoft.net/file/623668 Shize Li, Jing Liu, Guiping Yang, Gang Wei, HaiJun Su

## Figures and Tables

**Table 1.** Information for samples used in molecular phylogenetic analyses in this study (/ = not available).

ID	Species	Localities	Voucher	GenBank accession number		
				16S	COI	Cytb
1	*Brachytarsophrysqiannanensis* sp. nov.	China: Libo County, Qiannan Autonomous Prefecture, Guizhou	CIB LB20210806053	OK104099	OK104052	OK127913
2	*Brachytarsophrysqiannanensis* sp. nov.	China: Libo County, Qiannan Autonomous Prefecture, Guizhou	CIB LB20210806054	OK104100	OK104053	OK127914
3	*Brachytarsophrysqiannanensis* sp. nov.	China: Libo County, Qiannan Autonomous Prefecture, Guizhou	CIB LB20210806055	OK104101	OK104054	OK127915
4	*Brachytarsophrysqiannanensis* sp. nov.	China: Libo County, Qiannan Autonomous Prefecture, Guizhou	CIB LB20210806056	OK104102	OK104055	OK127916
5	*Brachytarsophrysorientalis*	China: Jiulianshan Nature Reserve, Longnan County, Jiangxi	SYS a004225	/	MT162625	MT162650
6	*Brachytarsophrysorientalis*	China: Jiulianshan Nature Reserve, Longnan County, Jiangxi	SYS a004228	/	MT162628	MT162653
7	*Brachytarsophrysorientalis*	China: Jiulianshan Nature Reserve, Longnan County, Jiangxi	SYS a004226	/	MT162626	MT162651
8	*Brachytarsophrysorientalis*	China: Jiulianshan Nature Reserve, Longnan County, Jiangxi	SYS a004486	/	MT162629	MT162654
9	*Brachytarsophrysorientalis*	China: Jiulianshan Nature Reserve, Longnan County, Jiangxi	SYS a005451	/	MT162632	MT162655
10	*Brachytarsophrysorientalis*	China: Jiulianshan Nature Reserve, Longnan County, Jiangxi	SYS a004227	/	MT162627	MT162652
11	*Brachytarsophrysorientalis*	China: Huboliao Nature Reserve, Nanjing County, Fujian	SYS a003340	/	MT162624	MT162649
12	*Brachytarsophrysorientalis*	China: Gutian Township, Shanghang County, Fujian	SYS a003249	/	MT162623	MT162648
13	*Brachytarsophryspopei*	China: Taoyuandong Nature Reserve, Yanling County, Hunan	SYS a001864	KM504256	MH406361	MH407191
14	*Brachytarsophryspopei*	China: Taoyuandong Nature Reserve, Yanling County, Hunan	SYS a001865	KM504257	MT162620	MT162645
15	*Brachytarsophryspopei*	China: Taoyuandong Nature Reserve, Yanling County, Hunan	SYS a001866	KM504258	MT162621	MT162646
16	*Brachytarsophryspopei*	China: Jinggang Shan, Jiangxi	SYS a004209	MK524124	MK524155	/
17	*Brachytarsophrysplatyparietus*	China: Duodihe, Dayao county, Yunnan	SYS a005919	/	MT162633	MT162656
18	*Brachytarsophrysplatyparietus*	China: Mt. Jinzhong, Longlin County, Guangxi	SYS a002236	/	MT162622	MT162647
19	*Brachytarsophrysplatyparietus*	China: Mt. Fanjing, Tongren City, Guizhou	YPX43968	/	MT162644	MT162667
20	*Brachytarsophrysplatyparietus*	China: Mt. Mopan, Xinping County, Yunnan	SYS a007774	/	MT162634	MT162657
21	*Brachytarsophrysplatyparietus*	China: Mt. Mopan, Xinping County, Yunnan	SYS a007775	/	MT162635	MT162658
22	*Brachytarsophrysplatyparietus*	China: Mt. Mopan, Xinping County, Yunnan	SYS a007776	/	MT162636	MT162659
23	*Brachytarsophrysplatyparietus*	China: Mt. Mopan, Xinping County, Yunnan	SYS a007777	/	MT162637	MT162660
24	*Brachytarsophrysplatyparietus*	China: Yilong Township, Shiping County, Yunnan	SYS a007790	/	MT162638	MT162661
25	*Brachytarsophrysplatyparietus*	China: Yumen Township, Yanbian County, Sichuan	SYS a007853	/	MT162639	MT162662
26	*Brachytarsophrysfeae*	China: Jingdong County, Yunnan	SYS a003912	MH406899	MH406362	MH407192
27	*Brachytarsophrysfeae*	China: Jingdong County, Yunnan	SYS a003913	/	MH406363	MH407193
28	*Brachytarsophrysfeae*	China: Huangcaoling, Yunnan	KIZ046706	KX811810	KX812056	/
29	*Brachytarsophryschuannanensis*	China: Zihuai Township, Hejiang County, Sichuan	SYS a004926	MH406901	MT162630	/
30	*Brachytarsophryscarinense*	Thailand: Doi Chiang Dao, Chiang Mai	K3001	KR827713	KR087626	/
31	*Brachytarsophryscarinense*	Thailand: Omkoi, Chiang Mai	KIZ024170	/	MT162640	MT162663
32	*Brachytarsophryscarinense*	Thailand: Mae Surin NP., Mae Hong Son	KIZ024429	/	MT162641	MT162664
33	*Brachytarsophryscarinense*	Thailand: Thong Pha Phum, Kanchanaburi	KIZ024640	/	MT162642	MT162665
34	*Brachytarsophrysintermedia*	Vietnam: Krong Pa, Gia Lai	ROM 23794	/	MT162643	MT162666
35	*Atympanophrysshapingensis*	/	CIBSC2011102004	JX458090	JX458090	JX458090
36	*Panophrysomeimontis*	China: Hongya County, Sichuan	MO-HY130601	KP728257	KP728257	KP728257

**Table 2.** Measurements of the adult specimens of *Brachytarsophrysqiannanensis* sp. nov. Units are given in mm. See abbreviations for the morphological characters in Materials and Methods section.

**Measurement**	**Male (n = 1)**	**Females (n = 3)**	
		Range	Mean ± SD
SVL	70.1	80.1 – 84.9	82.5 ± 2.4
HDL	18.9	20.6 – 24.8	22.0 ± 2.4
HDW	32.2	34.4 – 38.8	36.5 ± 2.2
SL	8.1	9.0 – 10.3	9.6 ± 0.7
IND	7.8	7.8 – 8.4	8.1 ± 0.3
IOD	9.4	9.2 – 11.3	10.5 ± 1.1
UEW	4.1	5.4 – 6.0	5.6 ± 0.3
ED	5.9	6.2 – 7.2	6.7 ± 0.5
TYD	4.1	3.0 – 4.1	3.7 ± 0.6
LAL	29.5	32.8 – 34.2	33.5 ± 0.7
LW	6.4	6.0 – 6.5	6.3 ± 0.2
THL	29.6	28.5 – 34.8	31.7 ± 3.2
TL	29.4	29.4 – 31.4	30.3 ± 1.0
TW	10.3	9.7 – 10.6	10.2 ± 0.5
TFL	41.9	42.7 – 44.8	43.6 ± 1.1
FL	32.6	31.8 – 32.3	32.0 ± 0.3
